# Working a second job: Cell adhesion proteins that moonlight in the nucleus

**DOI:** 10.3389/fcell.2023.1163553

**Published:** 2023-04-24

**Authors:** Amanda Haage, Archana Dhasarathy

**Affiliations:** Department of Biomedical Sciences, University of North Dakota, Grand Forks, ND, United States

**Keywords:** cell adhesion, extracellular matrix, mechanosensing, chromatin, transcription

## Abstract

Cells are adept at sensing changes in their environment, transmitting signals internally to coordinate responses to external stimuli, and thereby influencing adaptive changes in cell states and behavior. Often, this response involves modulation of gene expression in the nucleus, which is seen largely as a physically separated process from the rest of the cell. Mechanosensing, whereby a cell senses physical stimuli, and integrates and converts these inputs into downstream responses including signaling cascades and gene regulatory changes, involves the participation of several macromolecular structures. Of note, the extracellular matrix (ECM) and its constituent macromolecules comprise an essential part of the cellular microenvironment, allowing cells to interact with each other, and providing both structural and biochemical stimuli sensed by adhesion transmembrane receptors. This highway of information between the ECM, cell adhesion proteins, and the cytoskeleton regulates cellular behavior, the disruption of which results in disease. Emerging evidence suggests a more direct role for some of these adhesion proteins in chromatin structure and gene regulation, RNA maturation and other non-canonical functions. While many of these discoveries were previously limited to observations of cytoplasmic-nuclear transport, recent advances in microscopy, and biochemical, proteomic and genomic technologies have begun to significantly enhance our understanding of the impact of nuclear localization of these proteins. This review will briefly cover known cell adhesion proteins that migrate to the nucleus, and their downstream functions. We will outline recent advances in this very exciting yet still emerging field, with impact ranging from basic biology to disease states like cancer.

## 1 Introduction

**FIGURE 1:**
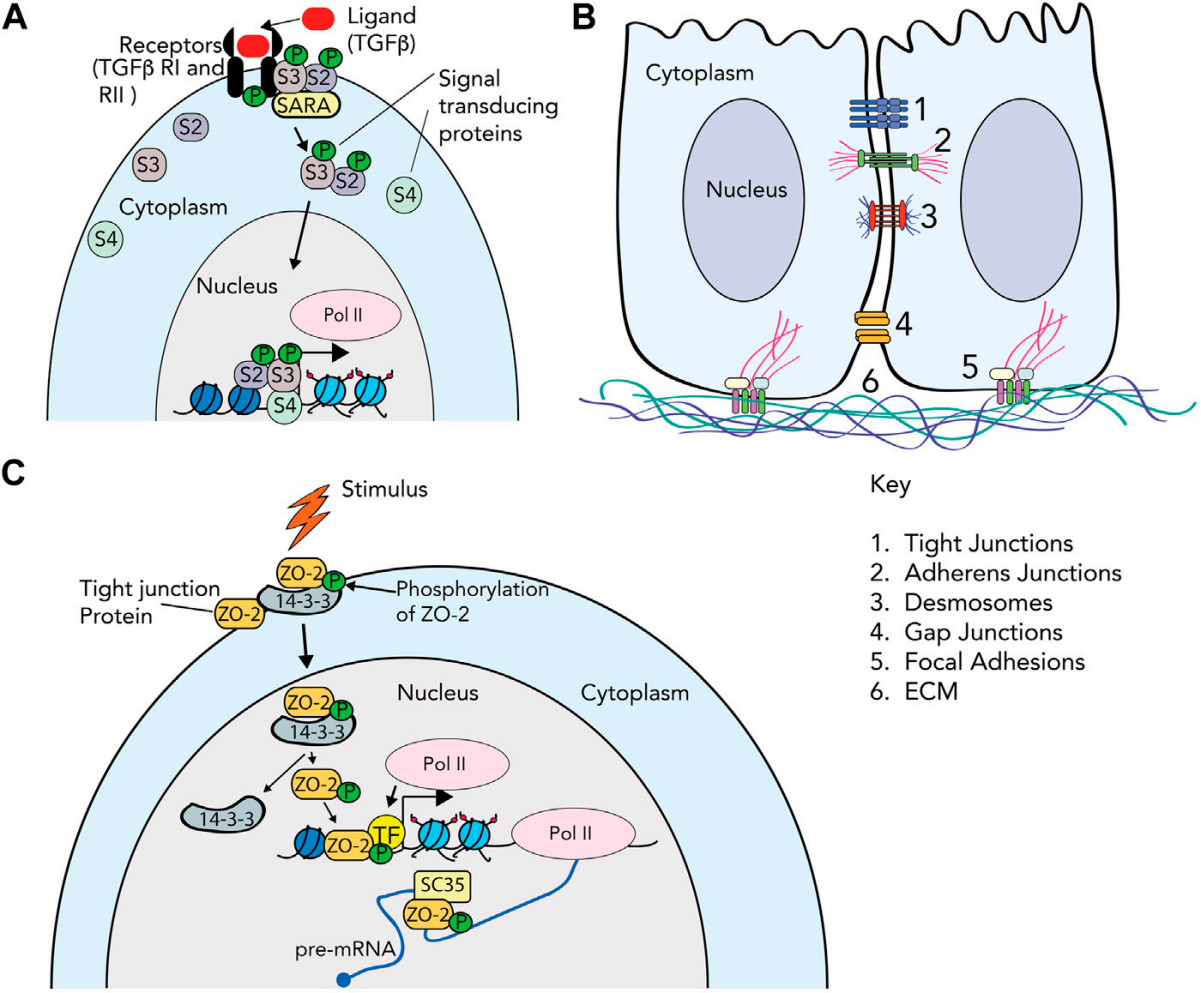
Cell attachment and cytoskeletal protein complexes. **(A)** *TGF-β signaling as an example of the classical signaling cascade*. The TGF-β ligand is recognized by specific receptors (TGF-βRII) on the cell membrane, which induces phosphorylation of TGF-βRI. Once TGF-βRI is activated, the binding of R-SMAD (receptor SMAD) proteins to the receptor is mediated by SARA (the SMAD anchor for receptor activation). The R-SMAD proteins (Smad2/S2 and Smad3/S3 in this example) are phosphorylated, and associate with co-Smad (Smad4/S4). The Smad proteins then transduce the signal to the nucleus by associating with chromatin, and activating or repressing gene expression. **(B)** *Cell junctions*. The cartoon depicts 2 cells, with cytoplasm and nucleus, and the multiple protein complexes that comprise (1) tight junctions; (2) adherens junctions; (3) desmosomes; (4) gap junctions; (5) focal adhesions; and (6) extracellular matrix. **(C)** *Signal transduction directly through cell adhesion proteins.* In this example, the ZO-2 protein in tight junctions is phosphorylated by SRPK1, and subsequently bound by the 14-3-3 protein and enters the nucleus. Inside the nucleus, ZO-2 interacts with transcription factors (TF) such as TEAD or SAF-b to alter gene transcription. It also associates with splicing protein SC-35 to regulate splicing. Other roles for various cell adhesion proteins that enter the nucleus such as telomere maintenance, splicing and chromatin structure are discussed in the text.

The basic steps of cell signaling are fundamental to any introductory biology course. An external signal is received by a cell receptor that transmits this signal to a variety of secondary messengers; which in turn relay the signal to the appropriate cellular address to elicit the required response. This is illustrated by the simplified version of TGF-β signaling presented in Figure 1A, discussed in more depth in the next section. We can classify these changes as short-term responses, like biochemical changes in enzyme activities, or long-term responses, like sustained changes in gene transcription or chromatin modifications. Cell signaling when viewed in the classical, reductionist sense, i.e., a lone signal, a single receptor, and a linear stepwise signal transduction pathway, conveys an easily readable system of cause and effect (Figure 1A).

In reality, cells are incredibly complex, and our understanding of their ability to interpret and respond to stimuli in their environment is constantly evolving. Cross-talk between multiple pathways responding to the same signal, and functional redundancy of signaling pathways are often the norm. The linear transduction steps seen in textbook diagrams are more accurately envisioned as complex spider webs that intersect, converge, and touch every metaphorical corner of the cellular environment. How cells make decisions, as interpreted by their observational outputs in response to a world full of inputs, is an area of increasing interest, and one that is marked by significant collaboration between classical biology and computational modeling. As our understanding of cell signaling has evolved, so too has the variety of what we understand as a “signal” or input that the cell takes into consideration.

While we remain limited by what we can observe, advances in microscopy, biochemical assays, genetic perturbation, high throughput genomic sequencing, etc., fine tune our observational tool-kit constantly. These tools help challenge our assumptions of what cells or even individual proteins can do. For example, use of high-resolution time-lapse microscopy allows us to track single proteins, and gene tagging allows us to observe where these proteins go. These systems give us the opportunity to observe proteins that traditionally function at the cell membrane as the “glue” that helps cells adhere to each other, or to the extracellular matrix, in novel localization patterns in the nucleus. These include proteins that make up focal adhesions, adherens junctions, tight junctions, gap junctions, and desmosomes (Figure 1B and Table 1). These cell adhesion proteins, which typically participate in either signal reception and/or short-term responses, are increasingly recognized as also being localized in the nucleus (Figure 1C) and directly participating in gene regulation, a classic long-term response, without the need for intermediary signal transducers (Hervy et al., 2006; Zuleger et al., 2012; Zheng and Jiang, 2022). This observation further breaks down the division of labor between molecules that function at relatively short versus long timescales.

**TABLE 1:** Summary of Cell adhesion proteins and their function in the nucleus.

Protein	Known function	Function in nucleus, if any	Mechanism(s) regulating nuclear translocation, if known	Function in development and/or disease states
3.1. Cell-ECM focal adhesion proteins				
***Talin***	Component of Focal adhesions. Activates B-integrin receptors; transmits mechanical stimuli to the actin cytoskeleton.	Depletion changes gene transcription. Effect on chromatin unknown but TLN1 interactions with Histone H4 and other chromatin proteins observed Da Silva et al. (2022).	Unknown. Could potentially be the FERM domain, similar to FAK?	Unknown
***FAK***	Non-receptor tyrosine kinase; component of FA	Binds to and regulates several transcription factors, including GATA4 Lim et al. (2012), p53 Lim et al. (2008), Runx1 Canel et al. (2017), and c-Jun Griffith et al. (2021) to control gene expression	NLS in the FERM domain of FAK Lim et al. (2008).	Overexpressed in many cancers Zhou et al. (2019). Nuclear function in cancer unclear.
***Integrins***	Transmembrane proteins involved in several cellular functions (adhesion, migration, immune system, proliferation, and cell differentiation)	α4β1 integrin drives histone H3 methylation (H3K9me2/3) through the methyltransferase G9a Zhang et al. (2016). Additionally, integrins can bind to nuclear scaffold protein Laminin Zhang et al. (2016) and contribute to the organization of the nucleus.	Unknown	Implicated in tumor formation, metastasis, autoimmune disease, infection and inflammation Fagerholm. (2022). Nuclear role in disease state unknown.
***Paxillin***	Focal adhesion protein; also maintains cytoskeleton.	Binds to mRNA binding protein, polyadenylation binding protein1 (PABP1) to enable nucleo-cytoplasmic export of mRNAs; enhances AR Miedlich et al. (2017)and ERK-mediated Sen et al. (2010) transcription by binding to chromatin.	NLS potentially in LIM domain Dong et al. (2009).	Implicated in nervous system, cardiac, and muscle development, and in oxidative stress, and several cancers Lopez-Colome et al. (2017). Nuclear function in disease unknown.
***ICAP-1***	ICAP1 is a phosphotyrosine binding (PTB) domain-containing protein that interacts with β1 integrin cytoplasmic domain	unknown	NLS (1MFRKGKKRHS10) Draheim et al. (2017)	Osteoblast proliferation and bone mineralization Bouvard et al. (2007). Nuclear function in disease unknown.
***Zyxin***	Phosphoprotein sensitive to mechanical stress; colocalizes with integrin receptors at cell- ECM attachment region.	Regulates gene transcription by interaction with transcription factors NMP4 Janssen and Marynen. (2006) and HNF1-beta Choi et al. (2013).	Has NES that restricts it to cytoplasm Nix and Beckerle. (1997). No traditional NLS observed, nuclear localization mechanism unknown Wang et al. (2019).	Multiple cancers Kotb et al. (2018), platelet biogenesis Yan et al. (2021). Nuclear function in disease unknown.
3.2 Cell-cell adhesion proteins				
***T-cadherin***	T-cadherin, (Cadherin 13, H-cadherin) lacks the transmembrane and cytoplasmic domains of other cadherins. Found anchored to plasma membrane.	Binds centrioles, potential role in cell cycle Andreeva et al. (2009)	Unknown	Upregulated in vascular proliferative disorders Rubina et al. (2021) and deregulated in many cancers Wyder et al. (2000). Muscle regeneration Tanaka et al. (2019). Nuclear function in disease unknown.
***E-cadherin***	Transmembrane glycoprotein involved in calcium-dependent cell adhesion; key structural component of adherens junctions.	C-terminal fragment of E-cadherin binds to DNA in complex with p120 Ferber et al. (2008) and modulates the p120-Kaiso-mediated signaling pathway.	Unknown	Inhibits Wnt signaling-dependent cancer stem cell phenotype Su et al. (2015); vascularization, and carcinogenesis Schneider and Kolligs. (2015). Nuclear function in disease unknown.
3.2 Cell-cell adhesion proteins				
***Zonula occludens (ZO) proteins***	Tight junction protein; membrane-associated guanylate kinase.	ZO-2 associated with heterogeneous nuclear ribonucleoprotein scaffold attachment factor-B (SAF-B) Traweger et al. (2003), which is involved in chromatin remodeling and RNA splicing Renz and Fackelmayer. (1996); Nayler et al. (1998). ZO-2 also associated with splicing protein SC-35Islas et al. (2002). ZO-2 recruitment of transcription factor TEAD to genes observed in sparse cultures Gallego-Gutierrez et al. (2021); González-Mariscal et al. (2022).	ZO-1: unknown; Potentially 1 or more NLSs located in N-terminus of ZO-2 Quiros et al. (2013).	Inflammation, gastrointestinal and liver cancer, asthma Bhat et al. (2018); Sugita and Kabashima. (2020). Nuclear function in disease unknown.
			ZO-2 has multiple serines that can be phosphorylated. Its nuclear localization signal is protected by 14-3-3 proteins Amaya et al. (2019). ZO-2 phosphorylation by PKCε and WNK4 regulate localization to tight junctions Amaya et al. (2019); González-Mariscal et al. (2022), and SRPK1 phosphorylation is required to enter the nucleus Quiros et al. (2013).	
***Connexin 43***	Transmembrane protein component of gap junctions.	Directly binds to N-cadherin promoter, interacts with Pol II to regulate transcription Kotini et al. (2018)	Exact NLS unknown; carboxy tail region localizes Connexin-43 to nucleus Dang et al. (2003).	Developmental disorders including neurological and heart diseases; Cancer Martins-Marques et al. (2019). Nuclear function in disease unknown.
***Desmoplakin***	Component of desmosomes.	Appears to bind to telomeres and involved in maintenance of telomeres Li et al. (2019).	Unknown	Cardiomyopathy Yuan et al. (2021); deregulated in cancer Najor. (2018). Nuclear function in disease unknown.
***Plakophilin***	Armadillo repeat-containing protein; component of desmosomes.	Interacts with ssDNA in nucleus Sobolik-Delmaire et al. (2010).	Unknown	Mutated in Ectodermal dysplasia/skin fragility syndrome; skin and heart disease, cancer Hofmann. (2020). Nuclear function in disease unknown.
***Plakoglobin***	Armadillo repeat containing protein and a paralog of β -catenin. Component of both the adherens junctions and desmosomes.	Binds p53 (in both nucleus and cytoplasm) and to gene promoter of 14-3-3-sigma protein in nucleus with p53 Aktary et al. (2013).	Unknown	Nuclear Plakoglobin involved in cardiomyopathy Lombardi et al. (2011). Nuclear function in disease unknown.

In this review, we will discuss recent advances in what we understand as signals in the cellular environment, and highlight the diversity of cell adhesion proteins that may have novel roles in the nucleus in more detail. It is important to note that the molecules that participate in more recently characterized forms of signaling, such as mechanosensing (Holle and Engler, 2011; Chen et al., 2017), are the very same ones that may have an expanded role in gene or epigenetic regulation. In this review, we will describe a few of the cell adhesion proteins that have so far been recognized to shuttle between nucleus and cytoplasm, and their putative roles in the nucleus. We will briefly touch on the implications of these findings in disease states, and technological advances that would enable further study of these cell adhesion structural proteins. When filtering through the vast array of possible cell signaling therapeutic targets, something that acts with a high level of redundancy and mechanistic diversity may be of great value.

## 2 Mechanosensing additions to classical cell signals

The classical view of cell signaling was solely understood in terms of chemical environment, viewing the extracellular matrix (ECM) and/or adherent substrates as merely necessary for anchorage-dependent growth. Hormones, cytokines, interleukins, growth factors, etc. that circulated in the bloodstream or extracellularly in tissues, and that could be easily added to laboratory culture conditions, dominated the cell signaling field. One example of this classical view (Figure 1A) is the TGF-β signaling pathway [reviewed in (Massague, 1998)]. In the canonical pathway, ligand (TGF-β) binding induces the oligomerization of the serine/threonine kinase receptors, TGF-β receptor type II and I. The Type II receptor subsequently phosphorylates the TGF-β type I receptor, which in turn phosphorylates and activates receptor-regulated SMAD proteins (R-SMADs). In the canonical pathway, phosphorylated Smad2 and Smad3 enter the nucleus in a complex with Smad4, where they bind to cognate DNA binding sites to regulate gene expression (Massague, 1998). It is of note that other non-canonical pathways of TGF-β signaling exist, which are beyond the scope of this mini-review.

While these classical signal transduction pathways remain important, a major sub-discipline took hold in the late 20th century exploring how cells responded to mechanical and physical forces. For instance, the shear stress induced during blood flow; or mechanical forces on joints during exercise. In the early 21st century, foundational work showed that stem cells can differentiate based on substrate stiffness alone, and that cells can migrate towards regions of increased rigidity (Engler et al., 2006), thereby birthing the field of mechanobiology.

Supported by technological advancements such atomic force microscopy (Alcaraz et al., 2018) or hydrogels that can be tuned to various stiffnesses (Fu et al., 2019), it is now well established that cells can sense and respond to aspects of their physical environment. The classic mechanosensing apparatus starts at the cell membrane with adhesion receptors. Tension or force is transmitted across, and therefore sensed by, integrins (Humphrey et al., 2014) in the case of cell-ECM adhesion, or cadherins (Yap et al., 2018) in the case of cell-cell adhesion, to intracellular adaptor proteins that mediate changes in cytoskeleton organization. A major such adaptor, Talin, acts as the direct converter between forces sensed across these adhesions and intracellular signaling, as it undergoes modular conformational change based on mechanical load (Yao et al., 2016). Domains in Talin’s rod region sequentially unfold based on the amount of force across the protein, acting as a spring and opening cryptic binding sites for Vinculin (Yao et al., 2014), another adaptor protein associated with cell-ECM adhesion, to initiate intracellular signaling changes.

Downstream, changes in substrate stiffness have been associated with a variety of short- and long-term behaviors. When cells were seeded on collagen-coated polyacrylamide substrates, they were shown to be capable of detecting substrate stiffness, and changing direction towards the area of increased stiffness (Lo et al., 2000). This phenomenon describing the directional migration of cells was termed *durotaxis* (Lo et al., 2000), which involves both mechanosensing and mechanotransduction of the signal to elicit cellular migration. The composition of the focal adhesion (FA) complex, including FA proteins Integrins, Talin and Vinculin are altered in response to mechanical cues, and modify the actin/myosin cytoskeleton as well as trigger signaling cascades to induce gene expression changes. Some examples are the Rho (Berrier et al., 2002) and ROCK (Zhou et al., 2013) pathways, as well as activation of the YAP/TAZ transcription factors (Totaro et al., 2018; Damkham et al., 2022). Cells can also upregulate or downregulate enzymatic activity (Haage and Schneider, 2014) based on substrate stiffness. Overall, mechanobiology has revolutionized cell signaling as a discipline that considers the input of both the physical and chemical environment.

## 3 Cell adhesion proteins as gene regulators

Concurrent with our increased understanding of the range and multiplicity of inputs a cell receives at any given moment, is our recognition of cellular responses to these inputs. In the classical view of signaling discussed above, a clear division of labor exists between proteins that we think of as responsible for each. For instance, there are cell surface receptors that recognize a signal, and distinct proteins downstream of the receptors that convey the signal to the nucleus (Figure 1A). Transcription factors in the nucleus downstream of the receptor bind to cognate DNA sites at gene promoters, and activate or repress genes that help cells adapt or respond to the stimulus. Transcription is assisted by epigenetic changes, a term encompassing heritable alterations in DNA methylation, histone modification, chromatin accessibility and non-coding RNAs. Epigenetic mechanisms allow both short-term gene regulation, but also function as bookmarks for long-term conservation of these responses (Allis and Jenuwein, 2016). The proteins that are the readers, writers and erasers of these epigenetic changes are well documented as primarily nuclear proteins (Tarakhovsky, 2010). In addition to transcription, epigenetic proteins are also involved in other mechanisms such as alternative splicing (Warns et al., 2016). Interestingly, accumulating evidence documents numerous instances where cytoskeletal or adhesion-associated proteins are found in the nucleus with possible roles in epigenetics and gene regulation (Hervy et al., 2006; Zuleger et al., 2012; Zheng and Jiang, 2022). When we accept redundancy, cross-talk, and complexity as the norm, it is easy to perceive this breakdown of labor division as the next major development in the cell signaling world. In this mini-review, we will highlight a few instances of the cell-ECM and cell-cell adhesion proteins known to function in the nucleus.

### 3.1 Cell-extracellular matrix, and focal adhesion proteins in the nucleus

Focal adhesions (FAs) are large protein complexes connecting the signals received from the ECM to the cytoskeletal machinery found within the cell (Burridge, 2017). The complexes center on integrin receptors that bind a variety of ECM proteins outside the cell, and a number of adaptor proteins and secondary messengers on their short cytoplasmic tails inside the cell. These adaptors form the direct connection to the cytoskeleton (Burridge, 2017; Byron et al., 2022). As mentioned previously, FAs are also major sites of mechanosensing, so the signals received from the ECM range from chemical to physical. A link between integrins and nuclear functions has been discussed in the literature, but mostly limited to indirect interactions. Nuclear size and positioning remain a major limit to where and how cells can migrate (Madrazo et al., 2017), a process controlled by signaling feedback between cells and the ECM mediated by integrins (Wagh et al., 2021). In addition, signaling downstream of integrin receptors has been shown to be regulated by histone methyltransferases acting outside of the nucleus on the FA proteins associated with their cytoplasmic tails. Conversely, Integrin α4β1 adhesion to Laminin-1 leads to altered histone methylation through interaction with the G9a methyltransferase, and nuclear stiffness in lymphocytes (Zhang et al., 2016). Although integrins seem restricted to having only indirect effects on nuclear function, their FA binding partners may have more direct roles.

There are 24 unique integrin heterodimers that function with a range of specificity across cell types (Takada et al., 2007). Despite this variety, many of these integrins converge on the same few adaptor proteins at the heart of their FA complexes, building hubs of signaling and scaffolding to the rest of the cell. One of the most well-studied of these adaptors, and the classic immunofluorescent marker for FAs, is Paxillin. Within FAs, Paxillin is best known for its signaling roles, binding tyrosine kinases such as focal adhesion kinase (FAK), and other adaptors (Turner, 2000). This role as a signal hub remains consistent as Paxillin cycles between FAs and the nucleus (Ma and Hammes, 2018). Paxillin co-localizes with mRNA binding proteins and steroid receptors in the nucleus, its translocation spurred by phosphorylation downstream of androgen-receptor stimulated ERK signaling (Sen et al., 2010). It has also been shown to target promoter regions of genes regulated by those same steroid pathways (Ma and Hammes, 2018).

Alternatively, Zyxin is an adaptor protein localized to FAs that is more known for its Actin-binding and regulating role. As such, it has a significant role in mechanosensing [reviewed in (Wang et al., 2019)]. Several studies have examined Zyxin localization changes in response to mechanical force, showing that it shifts from FAs to newly forming actin stress fibers under force, and can be recruited to the nucleus in cells experiencing force (Nix and Beckerle, 1997; Yoshigi et al., 2005; Uemura et al., 2011). Force-dependent gene expression changes have been observed to coincide with Zyxin nuclear localization and it has been shown to bind transcription factors NMP4 (Janssen and Marynen, 2006) and HNF1-beta (Choi et al., 2013), but a direct line between nuclear Zyxin and gene expression has yet to be drawn. Talin-1 is another important adaptor protein, mediating both mechanical dependent signaling and binding to the Actin cytoskeleton. Recently, Talin**-**1 was shown to localize to the nucleus, bind chromatin, and when nuclear localization was enhanced with addition of a genetic nuclear localization signal, gene expression changes were observed (Da Silva et al., 2022).

In addition to their identified solo roles, many types of Integrins, Paxillin, Zyxin and Talin also interact with each other. Integrin binding to the extracellular matrix induces Paxillin phosphorylation, and downstream recruitment of other proteins including focal adhesion kinase (FAK) and signal transduction proteins like Rac and Rho [reviewed in (Lopez-Colome et al., 2017)]. Paxillin and Zyxin are both independently recruited to stress fibers through their LIM domains (Smith et al., 2013), but it is unknown whether they interact directly. Talin has been shown to dimerize and form a complex with Paxillin to assist integrin-mediated FA formation (Lu et al., 2022). A recent publication reported clusters of Talin and Zyxin at the plasma membrane, and 70% of the time co-localized with each other (Tsunoyama et al., 2021). However, despite the interplay between the FA proteins, it is yet unknown whether FA proteins also influence each other’s translocation to the nucleus, and subsequent downstream events. It is interesting to speculate that inducing shifts in nuclear localization of these proteins could significantly affect the nanostructure of FAs (Kanchanawong et al., 2010), and subsequently their participation in canonical functions at the membrane.

Together, a theme emerges where adaptor proteins, which are not quite as restricted as the transmembrane integrins, can balance their short-term response duties at FAs to possible long-term response duties in regulating gene expression in the nucleus. What causes the shift between the two, or whether it is just a redundancy in the downstream signaling effects, remains mostly an open question. Interestingly, in a major study demonstrating Zyxin’s translocation to the nucleus (Cattaruzza et al., 2004), Vinculin, another well-known mechanosensing adaptor and Actin binding protein, did not shuttle to the nucleus under the same conditions, suggesting some specificity of Zyxin to proteins that will participate in both Actin binding and nuclear transport. Additionally, several key signaling partners to these adaptors such as FAK (Griffith et al., 2021) and ICAP-1 (Draheim et al., 2017), have also been detected in the nucleus under certain conditions, possibly creating a specific nucleo-adhesome protein pool with specific functions to be explored (Byron et al., 2022).

### 3.2 Cell-cell adhesion proteins in the nucleus

Adhesion between cells manifests as a variety of structures, specialized by different functions (Figure 1B). Tight junctions form near the apical surface of epithelial cells to make effective barriers. Adherens junctions and desmosomes function comparably to FAs, connecting adhesion between adjacent cells to the rest of the cytoskeleton, and participate in mechanosensing. Adaptor α-catenin unfolds under force as it is transmitted through transmembrane cadherins, to convert to chemical secondary messaging by binding to Vinculin (Seddiki et al., 2018), similar to Talin, which can also interact with cadherins. Gap junctions mediate intercellular communication by directly shuttling small molecules between cells. Each type of cell-cell adhesion has a unique set of molecular components working together to regulate both short- and long-term cell behavior, which was previously thought to occur via these transduction pathways. However, increasing evidence suggests that many of these components have direct roles in the nucleus.

Some of the earliest evidence for cell adhesion proteins playing a role in long-term response in the nucleus comes from the cadherin family of proteins that form adherens junctions. This also represents a significant departure from the trend, in that classical cadherins are transmembrane receptors, instead of cytoplasmic adaptors, that seem to present this dual localization and role. A C-terminal fragment of E-cadherin has been shown to localize to the nucleus, usually regulated by a downstream effector like β-catenin (Zhao et al., 2019), p120 (Ferber et al., 2008), and presenilin-1 (Haas et al., 2005). At least one of these complexes has been shown to bind DNA directly, regulating promoter activity (Ferber et al., 2008). Keeping up with this trend, T-cadherin, a unique cadherin for its lack of transmembrane domain, also localizes to the nucleus and, surprisingly, centrioles in endothelial cells, where it may have a role in the cell cycle (Andreeva et al., 2009).

Together, transmembrane Claudins and their cytoplasmic adaptors, Zonula Occludens, form tight junctions that maintain overall tissue permeability and polarity (Otani and Furuse, 2020). While Claudins are implicated in a variety of traditional signaling cascades, Zonula Occludens-1 and Zonula Occludens-2 (ZO-1 & ZO-2) have both been shown to shuttle back and forth from the membrane to the nucleus through use of nuclear localization and export signals that make them unique among their protein family (Gottardi et al., 1996; Traweger et al., 2003). For ZO-1, nuclear accumulation appears to be linked to the maturity of the cell-cell contacts, where less confluent cultures retain more ZO-1 in the nucleus (Gottardi et al., 1996). The role of ZO-1 in the nucleus remains unknown. ZO-2 accumulates in the nucleus of actively proliferating cells where it co-localizes with transcription factors like TEAD (Gallego-Gutierrez et al., 2021)and histone deacetylases to regulate gene transcription (Traweger et al., 2003).

Components of Desmosomes were also observed in the nucleus. Desmoplakin binds to telomeres and is known to be involved in their maintenance (Li et al., 2019). Plakophilin interacts with ssDNA in nucleus (Sobolik-Delmaire et al., 2010), while Plakoglobin was shown to bind to and regulate the gene promoter of the 14-3-3-sigma protein in nucleus with p53 (Aktary et al., 2013). Lastly, the Carboxy tail of Connexin 43, a transmembrane gap junction protein, has been observed to enter the nucleus (Dang et al., 2003; Kotini et al., 2018), directly bind to the N-cadherin promoter and interact with Pol II to regulate gene transcription (Kotini et al., 2018). While Connexin is implicated in developmental disorders and cancer (Martins-Marques et al., 2019), whether the nuclear form is involved in disease progression remains unclear.

### 3.3 Crosstalk with cytoskeletal proteins in the nucleus

One potential way that cell adhesion proteins could operate to influence cellular processes in the nucleus is through their existing interactions with cytoskeletal proteins, several of which are known to have dual roles in the nucleus and cytoplasm. For example, Myosins, which are molecular motor proteins, are involved in chromatin dynamics and epigenetic mechanisms to regulate gene expression. Myosin directly binds to DNA through its cargo binding domain to regulate these functions (Fili et al., 2017). Similarly, Nuclear β-actin regulates enhancer function by influencing H3K27 acetylation levels (Mahmood et al., 2023); while association of nuclear Tubulin with chromatin was known as early as the 1980s (Menko and Tan, 1980). It is therefore easy to envision existing interactions with cell adhesion proteins being used for additional functions such as transcriptional regulation within the nuclear milieu.

## 4 Nuclear function of cell adhesion proteins and misregulation in disease

Despite such a plethora of cell adhesion proteins that were found to migrate to the nucleus (Table 1), little is known about how these proteins function in the nucleus, and whether these role(s) are altered during, and/or are relevant for, normal development and disease states. The nuclear function of three of the focal adhesion proteins, Talin-1, FAK, and Zyxin have been explored to some detail. For instance, a recent paper demonstrated that Talin-1 depletion altered gene expression and was copurified with the chromatin fraction of cells (Da Silva et al., 2022), but the direct result of this interaction is yet forthcoming. Importantly, the relevance of this nuclear localization of Talin-1 in both development and disease remains a key question.

Other proteins of FAs that have been documented to have direct effects on transcription include FAK, which has been demonstrated to bind transcription factors to control gene expression. Specifically, one study showed that FAK regulated expression of cytokine IL-33 by mediating chromatin accessibility of its enhancer element, thereby allowing transcription factor c-Jun to bind and regulate expression of IL-33 (Griffith et al., 2021). Zyxin, another FA protein, was observed to regulate gene transcription by interaction with transcription factors such as nuclear matrix protein 4 (NMP4/ZNF384) (Janssen and Marynen, 2006) and HNF1-beta (Choi et al., 2013). Zyxin is particularly interesting in that it appears to have a mechanosensory function and enters the nucleus in response to mechanical force stimulation [reviewed in (Wang et al., 2019)], whereas the stimulus for an interaction between FAK and chromatin remains unknown.

While stimuli for nuclear accumulation of proteins involved in cell-cell adhesion is not clearly defined, they may have a role in regulating cancer growth and invasiveness. Perhaps the most convincing evidence is for E-cadherin, nuclear staining of which was noted for multiple cancers (Chetty and Serra, 2008). Interestingly, nuclear expression is correlated with good prognosis in certain cancers (e.g., clear cell renal cancers), but not in others like pancreatic neuroendocrine tumors, which speaks to fine-tuning of its role in the nucleus, potentially through interactions with other proteins. This idea is supported by the finding that acetylation of nuclear E-cadherin prevented its interaction with β-catenin, thereby increasing tumor cell invasion (Su et al., 2015; Zhao et al., 2019). β-catenin is a key structural component of the cell-cell adhesion complexes through interaction with E-cadherin. In the presence of Wnt signaling, β-catenin can enter the nucleus and regulate gene expression through interaction with transcriptional proteins (Nusse and Clevers, 2017). This phenomenon of traditional secondary messengers directing the shuttling of cell adhesion proteins between the membrane and the nucleus might be more common than we think, and is worth investigating as part of the mechanism of regulation.

Another cell-cell adhesion protein, ZO-2, has an NLS that can be phosphorylated by different kinases with varying outcomes (Quiros et al., 2013; Gallego-Gutierrez et al., 2021; González-Mariscal et al., 2022). The phosphorylation of ZO-2 by SRPK1 kinase allows it migrate to the nucleus (Quiros et al., 2013) in association with 14-3-3 proteins (Amaya et al., 2019), where it can associate with transcription factor TEAD (Gallego-Gutierrez et al., 2021), as well as the heterogeneous nuclear ribonucleoprotein scaffold attachment factor-B (SAF-b) (Traweger et al., 2003). SAF-b binds specifically to DNA in the scaffold or matrix attachment region of the nucleus (Renz and Fackelmayer, 1996), and is also implicated in both transcription and splicing regulation (Nayler et al., 1998). ZO-2 might be guilty by association of having a role in these mechanisms as well. ZO-2 also co-localizes with the splicing protein SC-35 (Islas et al., 2002), reinforcing its link with splicing regulation. Other cell-cell adhesion proteins such as Paxillin and Connexin 43 have roles in gene transcription as well; Paxillin through ERK-mediated transcription (Sen et al., 2010) and Connexin through direct binding to DNA coupled with Pol II recruitment (Kotini et al., 2018) to the N-cadherin promoter. Other than transcription, nuclear functions such as nucleo-cytoplasmic export of mRNAs; in the case of Paxillin (Miedlich et al., 2017); or maintenance of telomeres, in the case of Desmoplakin (Li et al., 2019) have been noted in the literature. However, for the vast majority of cell adhesion proteins, it is unclear whether their presence and function in the nucleus directly impacts development or disease.

Interestingly, many of these cell adhesion proteins are themselves regulated during the epithelial-mesenchymal transition (EMT), a key driver of cell migration and invasion during development and cancer metastasis. During EMT, gap junctions, tight junctions, adherens junctions and desmosomes lose their integrity [reviewed in (Lamouille et al., 2014)], and EMT transcription factors like SNAIL decrease expression of adhesion proteins like E-cadherin, Occludin, and Claudins, while increasing classical TGFβ signaling (Dhasarathy et al., 2011). While the loss of expression of these cell adhesion proteins is often thought of in terms of loss of cell structure and motility, it might be important to inquire whether they also affect gene expression due to loss or gain of nuclear localization. Finally, matrix stiffness and mechanotransduction are now thought to be important in driving EMT-driven chemoresistance and metastasis (Wei et al., 2015; Rice et al., 2017; Fattet et al., 2020), and it is worth inquiring how cell adhesion proteins in the nucleus might be relevant to this process.

## 5 Conclusion

The importance of cell-cell adhesion proteins is underscored by the fact that several of these proteins are highly conserved across metazoan species. As far back as invertebrates (Drosophila and C. elegans), high conservation of cadherins and immunoglobulin superfamily (Ig-SF) proteins was observed (Black et al., 1990), but some cell-cell adhesion proteins (protocadherins, desmocollins, desmogleins) were found to have emerged later in evolution in the chordates (Black et al., 1990). Similarly, cell-matrix adhesion proteins were found to be conserved as far back as invertebrates; including integrins, laminins and proteoglycans (Black et al., 1990). Evolutionary analyses could also help address whether loss or gain of functional motifs, such as the LIM domain or NLS sequences in some of these proteins could mirror their ability to migrate to the nucleus. Interestingly, some novel integrins with no homologues in higher vertebrates were observed in *Drosophila* and other invertebrates (Black et al., 1990)), which might be important for specialized functions in these animals. Further, several vascular ECM proteins are absent in insects, presumably as a more sophisticated vasculature evolved in chordates (Black et al., 1990). Recent advances in genome-wide analysis techniques could answer questions raised by these types of evolutionary analyses, including whether cell adhesion proteins exhibit similar nuclear functions across species.

The burgeoning advances in technologies in recent years have enabled us glimpses of the complexity of cell signaling, cementing the idea that linear pathways in textbooks need to be reevaluated. Advances such as super-resolution and expansion microscopy have helped us understand the range of proteins that shuttle to and from the nucleus, even those that were previously thought of as solely structural in nature. However, a lot remains unknown, which could be addressed with some current technologies. For instance, the question of how these proteins decide between their structural role at the edge of the cell membrane, and their regulatory function in the innermost nuclear locale of the cell, and how they are transported into the nucleus can be answered by proteomics, to determine their interaction partners. The question of which genes are regulated by these proteins in the nucleus can be addressed by RNA-sequencing, ATAC-sequencing and ChIP-sequencing, while their mechanosensing role could be addressed with methods such as Hi-C that interrogates changes to the 3D genome architecture. An additional open question is what happens to FA and cell-cell adhesion protein structure and functionality once any of their constituent proteins shift to the nucleus. Does loss of these cell-cell adhesion or FA proteins result in weaker/smaller FAs or junctions, structures that are less capable of performing their normal functions like mechanosensing? The answers to these and additional questions, such as what conditions are optimal for their translocation, and why some signals trigger their translocation but not others, will prove of prime importance in understanding fundamental questions of how cells respond to their environment. Addressing these fundamental questions will aid in the development of biomarkers of when these processes are dysregulated in disease, as well as in the development of drug therapies targeting these proteins. The advent of these and other technologies, such as spatial transcriptomics, will help make the coming decade prove a fruitful one for further study of novel roles for cell adhesion proteins.
